# Elevated CA19-9 as the Most Significant Prognostic Factor in Locally Advanced Rectal Cancer Following Neoadjuvant Chemoradiotherapy

**DOI:** 10.1097/MD.0000000000001793

**Published:** 2015-11-13

**Authors:** Lu-Ning Zhang, Pu-Yun OuYang, Wei-Wei Xiao, Xin Yu, Kai-Yun You, Zhi-Fan Zeng, Rui-Hua Xu, Yuan-Hong Gao

**Affiliations:** From the Department of Radiation Oncology (L-NZ, P-YOY, W-WX, XY, Z-FZ, Y-HG), Department of Medical Oncology, Sun Yat-sen University Cancer Center, State Key Laboratory of Oncology in South China, Collaborative Innovation Center for Cancer Medicine, Guangzhou, Guangdong (R-HX) and Department of Oncology, The Second Affiliated Hospital of Sun Yat-sen University, Guangzhou, China (K-YY).

## Abstract

It remains controversial regarding the prognostic significance of carbohydrate antigen 19-9 (CA19-9) for locally advanced rectal cancer (LARC) (T3–4/N+) patients with neoadjuvant chemoradiotherapy (neo-CRT). And it is unknown whether CA19-9 can identify patients who may benefit from adjuvant chemotherapy.

Overall, 303 LARC patients with neo-CRT between 2004 and 2010 were recruited. Overall survival (OS), disease-free survival (DFS), distant metastasis-free survival (DMFS), and local recurrence-free survival across pretreatment CA19-9 were estimated by Kaplan–Meier method and Cox regression model.

In univariate analysis, elevated CA19-9 (>35 U/mL) was significantly correlated with poor OS (*P* = 0.003), DFS (*P* = 0.001), and DMFS (*P* = 0.039). Adjusting for the known covariates, CA19-9 was significantly associated with OS (HR = 1.86, 95% CI 1.03–3.34, *P* = 0.039) and DFS (HR = 1.74, 95% CI 1.08–2.80, *P* = 0.024). In the elevated CA19-9 subgroup, patients with adjuvant chemotherapy got much better OS (*P* < 0.001) and DFS (*P* = 0.016) than those without. In consideration of both CA19-9 and carcinoembryonic antigen (CEA), we found that patients with both elevated CA19-9 and CEA (>5 ng/mL) got the worst OS (*P* = 0.021) and DFS (*P* = 0.006), and significantly benefited from adjuvant chemotherapy in OS (*P* < 0.001) and DFS (*P* = 0.026).

Pretreatment CA19-9 level is a significant prognostic indicator in patients with LARC following neo-CRT. The addition of CA19-9 to CEA is valuable to discriminate the appropriate patients for adjuvant chemotherapy.

## INTRODUCTION

Tumor markers are useful tests in the management of patients with cancer and connote both minimal inconvenience and low financial expenses compared to endoscopic procedures and novel imaging techniques. Generally, tumor markers are not useful for diagnosis because of their low specificity and sensitivity, but they are useful tests in the follow-up of neoplastic patients. Moreover, several authors propose tumor markers as prognostic factors in different tumors.^1^ In recent years, a multiplicity of tumor markers have been proposed for colorectal cancer (CRC).^2–6^ According to current guideline recommendations, carcinoembryonic antigen (CEA) is the most important tumor marker with regard to individual prognosis, detection of recurrent disease, and on-treatment monitoring in all stages of CRC.^7–9^

For ameliorated prediction of prognosis and optimized surveillance of CRC patients, several other serum markers have been investigated. The availability in clinicopathologic investigation on colorectal carcinoma of carbohydrate antigen 19-9 (CA19-9) has been demonstrated in many reports,^10–16^ although its sensitive to detect colorectal carcinoma has been reported to be less than does CEA, and to this date its role for screening, staging, and treatment monitoring for CRC cannot be recommended due to insufficient data.^9^ However, the possible application of CA19-9 values in prognosis is still controversial. Several studies^11–13^ suggested that CA19-9 level was one of the best available prognostic indicators in advanced colorectal carcinoma. Especially, Nakagoe et al^10^ showed that elevated preoperative serum levels of CA19-9 may serve as a useful marker in identifying patients with node-negative CRCs at high risk for recurrence after surgery. Contradictory to the above findings, other studies^14–16^ showed that CA19-9 had no prognostic significance in advanced colorectal adenocarcinoma.

Furthermore, the significance of increase in both markers to predict the prognosis of the patients remains a problem for debate. Some studies reported that the combination of preoperative CEA and CA19-9 levels was useful for predicting the prognosis after surgery. Notably, Shibutani et al^17^ restricted patients in stage II CRC which may limit the application of their conclusion. Nozoe et al^18^ only recruited 103 patients with CRC which might make the results skewed.

More importantly, nearly all the above investigations drew their conclusions in CRC. However, it is known that aside from embryological, anatomical, and physiological differences between the colon and rectum, colon and rectal cancer seem to differ in oncogenesis.^19^ Thus, the aim of the present study was to elucidate the further clinicopathologic significance of increase in pretreatment serum CA19-9 and test the hypothesis that the combination of preoperative CEA and CA19-9 serum levels could be more sensitive and specific in prediction of survival in locally advanced rectal cancer following neoadjuvant chemoradiotherapy (neo-CRT).

## METHODS AND MATERIALS

### Patients

This retrospective study was approved by the Institutional Review Board at Sun Yat-sen University Cancer Center, and individual informed consent was waived given the anonymous analysis of routine data. A total of 303 patients undergoing preoperative neo-CRT followed by radical surgery at our center between October 2004 and December 2012 were recruited. Rectal carcinoma was clinically diagnosed based on abdominal and pelvic computed tomography (CT), magnetic resonance imaging (MRI), and endorectal ultrasound (ERUS). In our cancer center, ERUS is recommended for every patient for accurate T staging. Other examinations such as complete blood cell count and liver function tests were also conducted. All of the patients had pathologically-proven rectal carcinoma.

### Treatment

Radiotherapy was delivered to the whole pelvis at a dose of 46 Gy in 23 fractions, followed by a 4-Gy boost delivered to the primary tumor in 2 fractions for 5 weeks. The method of radiotherapy had been described in prior study.^20^

The main preoperative concurrent chemotherapeutic regimens were XELOX (oxaliplatin 100 mg/m^2^, d1 + capecitabine 1000 mg/m^2^ bid, po, d1–14), FOLFOX6 (oxaliplatin 85 mg/m^2^, d1 + leucovorin 400 mg/m^2^, d1 + 5-FU 400 mg/m^2^ iv, d1 followed by 2400 mg/m^2^ civ 46–48 hr) or Xeloda (capecitabine 1000 mg/m^2^ bid, po, d1–14).

Surgery was performed 6 to 8 weeks after the completion of preoperative CRT. All patients underwent radical proctectomy, including low anterior resection (LAR), abdominoperineal resection, and Hartmann's procedure.

Postoperative adjuvant chemotherapy was recommended for all patients, irrespective of the surgical pathological results, in accordance with National Comprehensive Cancer Network (NCCN) guidelines. However, only 218 patients received adjuvant chemotherapy, either XELOX or FOLFOX6, 4 weeks after surgery. The other 85 patients received no adjuvant chemotherapy owing to postoperative complications, poor overall performance status, or economical problem.

### Follow Up

Follow up was performed every 3 months for the first 2 years after whole treatment and every 6 months thereafter. Evaluations included complete blood cell count, liver function tests, serum CEA and CA-199 level tests, physical examination, and digital rectal examination at each visit. Chest radiography, abdominal and pelvic CT scanning, and colonoscopy were conducted every 6 months after surgery. Positron emission tomography (PET)/CT is not regularly recommended, although some patients prefer it due to its advantage in early detection of recurrence. The last follow up was completed in May 2015.

### Statistical Analysis

The primary endpoints were OS and DFS, which were defined as the time from completion of the whole treatment to death from any cause and to the first occurrence of either local or distant progression or of death in the absence of such an event, respectively. The secondary endpoints were DMFS and LRFS. Distant metastasis was identified as any recurrence outside of the pelvic cavity. Local recurrence was defined as any recurrence within the pelvic cavity or perineum.

OS, DFS, DMFS, and LRFS rates were estimated using the Kaplan–Meier method and the log-rank test. Multivariate analysis was performed using the Cox proportional hazards regression. Two-sided *P* < 0.05 was considered statistically significant. All statistical analyses were performed using SPSS software, version 20.

## RESULTS

### Patients

The median follow up was 42 months (range; 5–126 months). There were 14 cases (4.6%) of locoregional relapse, 64 cases (21%) of distant metastasis, and 60 cases (19.8%) of death, respectively. Six patients (1.98%) had both locoregional relapse and distant metastasis. The baseline characteristics of the 303 patients were listed in Table 1. The 3- and 5-year OS rates were 87.5% and 77.6%, and the 3- and 5-year DFS rates were 73.7% and 66.4%, respectively.

**TABLE 1:**
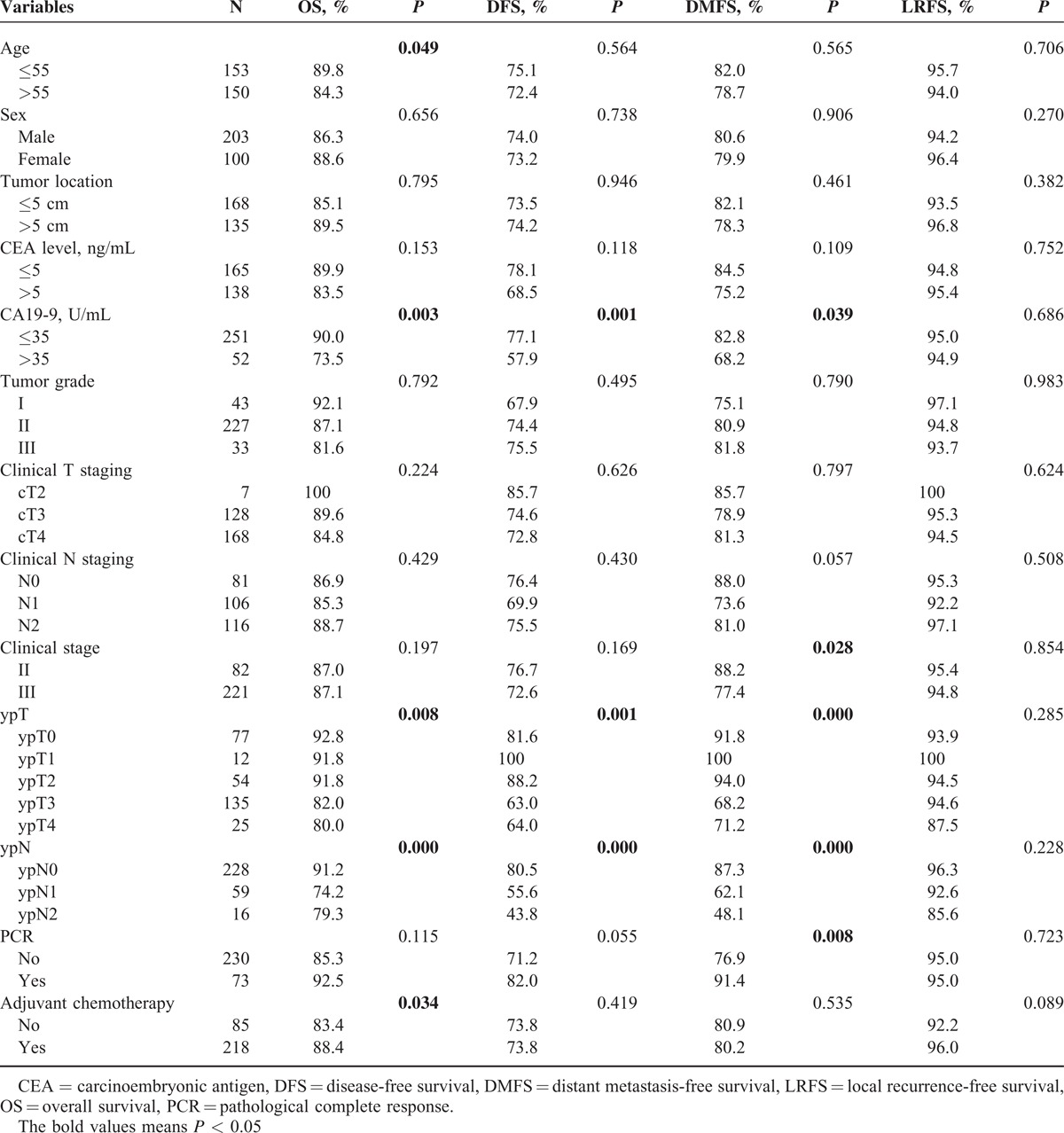
Influence of Different Variables on 3-Year OS, DFS, DMFS, and LRFS in Locally Advanced Rectal Cancer Following Neoadjuvant Chemoradiotherapy

Overall, 138 (45.5%) patients had an elevated CEA level (>5 ng/mL) and 52 patients (17.2%) with an elevated CA19-9 level (>35 U/mL). Thirty-eight patients (12.5%) had both high CEA and CA19-9 levels. The correlations between the preoperative CEA/CA19-9 levels and the clinicopathological parameters were shown in Table 2. ypT (*P* < 0.001) and ypN (*P* = 0.015) were associated with high CEA level, while only ypN (*P* = 0.009) was correlated with high CA19-9 level.

**TABLE 2:**
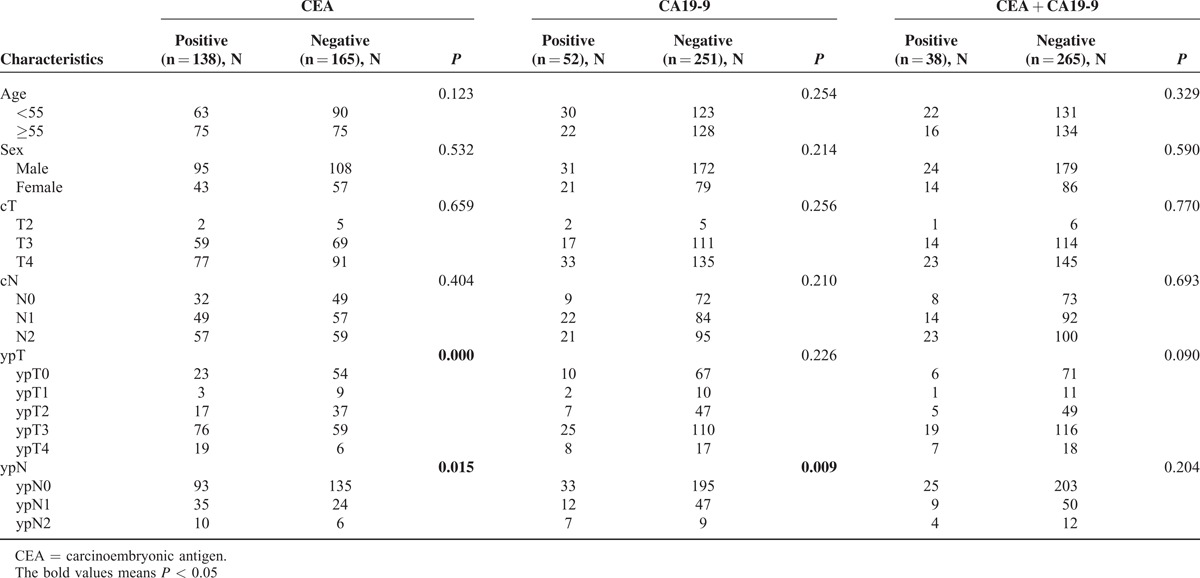
Association of CEA/CA19-9 With Different Factors in Patients With Locally Advanced Rectal Cancer Following Neoadjuvant Chemoradiotherapy

Among patients with both elevated CA19-9 and CEA, no relationship was found between clinicopathologic factors and both tumor markers (Table 2).

### CA19-9 Is Predictive of OS and DFS

**FIGURE 1:**
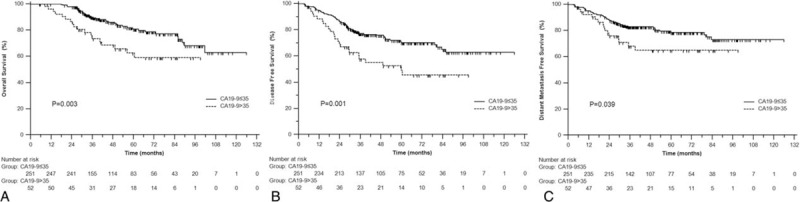
Overall survival (A), disease-free survival (B), and distant metastasis-free survival (C) for locally advanced rectal cancer patients with elevated CA19-9 and normal CA19-9.

**FIGURE 2:**
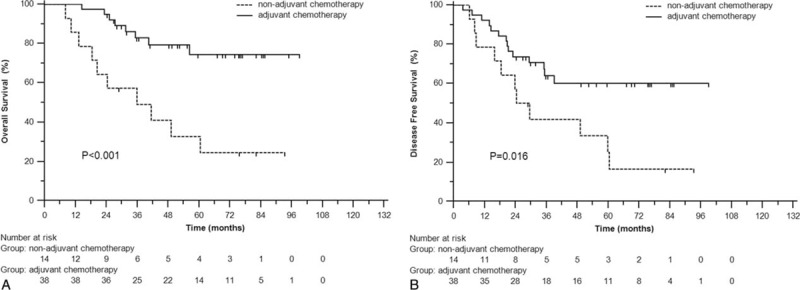
In patients with elevated CA19-9, the overall survival (A) and disease-free survival (B) of patients with and without postoperative adjuvant chemotherapy.

In univariate analysis, CA19-9 was significantly associated with poor OS (3-year 73.5% vs 90.0%, *P* = 0.003), DFS (57.9% vs 77.1%, *P* = 0.001), and DMFS (68.2% vs 82.8%, *P* = 0.039) (Figure 1A–C). Adjusting for the known covariates, patients with elevated CA19-9 were significantly correlated with OS (HR = 1.86, 95% CI 1.03–3.34, *P* = 0.039) and DFS (HR = 1.74, 95% CI 1.08–2.80, *P* = 0.024) (Table 3). Subgroup analysis indicated that in elevated CA19-9 (>35 U/mL) group, patients who underwent adjuvant chemotherapy got much better OS (*P* < 0.001) and DFS (*P* = 0.016) (Figure 2A and B), whereas in normal CA19-9 (<35 U/mL) group, no significant differences were observed in OS (*P* = 0.547) or DFS (*P* = 0.747).

**TABLE 3:**
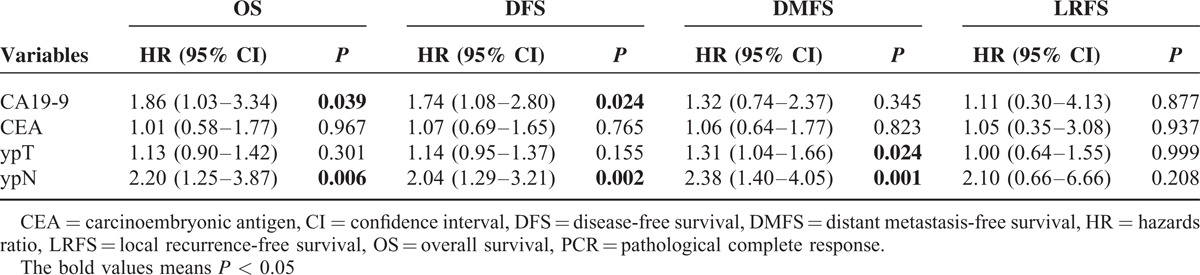
Multivariable Analysis of Different Variables on 3-Year OS, DFS, DMFS, and LRFS in Locally Advanced Rectal Cancer Following Neoadjuvant Chemoradiotherapy

### CEA Is Not Predictive of OS or DFS

Albeit CEA is a widely accepted prognostic factor in CRC, no significant differences were observed in OS (*P* = 0.153), DFS (*P* = 0.118), or DMFS (*P* = 0.109) in univariate analysis in the present study. And it remained nonsignificant after adjusting for the known covariates by multivariate analysis (Table 3).

### The Combination of CEA and CA19-9 Is Predictive of OS and DFS

**FIGURE 3:**
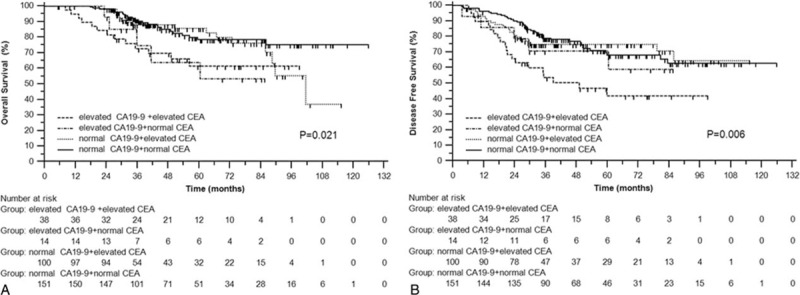
Overall survival (A) and disease-free survival (B) of group 1 (elevated CA19-9 + CEA), group 2 (elevated CA19-9 + normal CEA), group 3 (normal CA19-9 + elevated CEA), and group 4 (normal CA19-9 + CEA). CEA = carcinoembryonic antigen.

**FIGURE 4:**
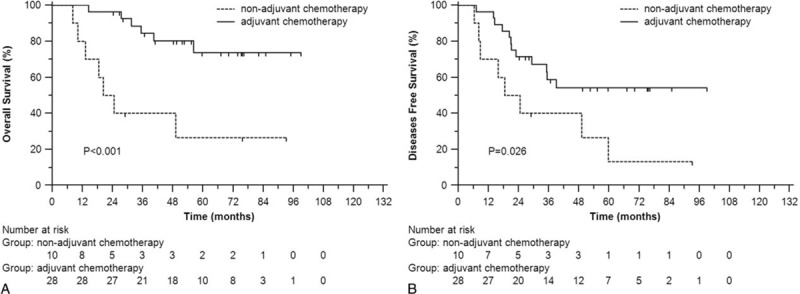
In patients with both elevated CEA and CA19-9, the overall survival (A) and disease-free survival (B) of patients with and without postoperative adjuvant chemotherapy. CEA = carcinoembryonic antigen.

We divided all the patients into 4 groups: group 1, elevated CA19-9 and elevated CEA; group 2, elevated CA19-9 and normal CEA; group 3, normal CA19-9 and elevated CEA; and group 4, normal CA19-9 and normal CEA. Consequently, the patients in group 1 got the worst OS (*P* = 0.021) and DFS (*P* = 0.006) (Figure 3A and B). Fortunately, adjuvant chemotherapy significantly improved OS (*P* < 0.001) and DFS (*P* = 0.026) for patients in group 1 (Figure 4A and B). However, patients in the other groups cannot benefit from adjuvant chemotherapy in OS (*P* = 0.295 for group 2; *P* = 0.720 for group 3; *P* = 0.484 for group 4) or DFS (*P* = 0.164 for group 2; *P* = 0.675 for group 3; *P* = 0.981 for group 4).

## DISCUSSION

In our study, ypT and ypN were associated with high CEA level, while only ypN was correlated with high CA19-9 level but no correlation was found between clinicopathologic factors and elevation of these 2 markers. In univariate and multivariate analyses, patients with elevated CA19-9 had significantly poorer OS and DFS but fortunately postoperative adjuvant chemotherapy could improve the survival of this subgroup of patients. Interestingly, elevated CEA was not significantly correlated with worse prognosis, while patients with both elevated CA19-9 and CEA got the worst OS and DFS and could benefit from postoperative adjuvant chemotherapy.

It is known that the elevated CEA and CA19-9 levels always represent heavy tumor load which can partly explain the relationship with pathological changes. Consistent with prior studies,^10–13^ we also found that patients with elevated CA19-9 had significantly worse OS and DFS. Of note, our study had the largest cohort of 303 locally advanced rectal cancer patients with neo-CRT. Inversely, Webb^14^ reviewed 78 CRC patients with elevated CA19-9 and found that this marker was not of prognostic significance. Morita et al^15^ analyzed 114 colorectal adenocarcinoma patients who underwent potentially curative surgery and emphasized that they could not find clinical significance to support the use of CA19-9 to predict the prognosis and detect recurrence of CRC. Obviously, the small sample size of these 2 studies greatly lowered the confidence of the findings. Giessen^16^ analyzed 256 rectal cancer patients but adopted the median value of 10.6 (U/mL) as the cut-off of CA19-9 level, which may obviously affect their statistical results. Given the poor prognostic impact of elevated CA19-9, we further investigated the role of adjuvant chemotherapy in the according strata. Interestingly, the subgroup analysis showed that in elevated CA19-9 group, adjuvant chemotherapy lead to a better OS and DFS.

CEA is a widely accepted prognostic factor in CRC.^7–9^ But the present study did not find any clinical significance of CEA in predicting prognosis in patients with locally advanced rectal cancer. This was highly consistent with the results of the study by Filella et al^11^ in which CEA showed no statistical significance. Similarly, Kouri et al^12^ also showed no prognostic value of serum CEA, albeit that CEA seems to be the best tumor marker for response prediction. Thus we assumed that the combination of preoperative CEA and CA19-9 might be more sensitive and specific in predicting survival. As presented in other studies,^21,22^ the sensitivity of CEA in our series was 45.3%, while the combination of CA19-9 with CEA increased the sensitivity to 57.8%. Furthermore, patients with both elevated CEA and CA19-9 showed the worst prognosis.

The most important finding in the present study is that patients with high CA19-9 only or both high CA19-9 and CEA can benefit from adjuvant chemotherapy. The molecular basis for this is poorly understood. But it is known that CA19-9 is an antigen expressed by the glycosylated extracellular MUC1 protein and plays an important role in cancer invasion by enhancing cell adhesion and promoting angiogenesis indirectly.^23^ This may partly explain why patients with elevated CA19-9 can get a better survival after receiving adjuvant chemotherapy. What is more, several studies had reported that rectal cancer has less microsatellite instability and fewer *BRAF* mutations than colon cancer does.^24–26^ Different gene expression profiles between colon and rectal cancer have been reported.^27,28^ So another hypothesis is that maybe in rectal cancer, CA19-9 plays a more important role than CEA does, whereas in colon rectal CEA is more important. These differences might contribute to the different effects of adjuvant chemotherapy in colon and rectal cancer.

As with any retrospective study, there is possibility of confounders and issues with missing data. But clinicopathologic and survival data were verified by review of individual patient record. Moreover, the treatment heterogeneity, especially the influence of adjuvant chemotherapy, was another limitation due to the retrospective design. But all included patients received standard management of neoadjuvant chemotherapy and TME as recommended. Of note, it was a limitation that the number of patients in this study made it unavailable to conduct subgroup analysis across tumor stage, for example.

To summarize, our study showed that the serum CA19-9 value functioned as a significant prognostic factor in neo-CRT-treated patients with locally advanced rectal carcinoma. Combination CEA and CA19-9 in sera can provide more powerful and useful information to predict prognosis. Importantly, patients with elevated CA19-9 alone or both CEA and CA19-9 elevation can benefit from adjuvant chemotherapy.
